# A flexibly shaped spatial scan statistic for detecting clusters

**DOI:** 10.1186/1476-072X-4-11

**Published:** 2005-05-18

**Authors:** Toshiro Tango, Kunihiko Takahashi

**Affiliations:** 1 Department of Technology Assessment and Biostatistics, National Institute of Public Health, 3–6 Minami 2 chome Wako, Saitama 351-0197 Japan

## Abstract

**Background:**

The spatial scan statistic proposed by Kulldorff has been applied to a wide variety of epidemiological studies for cluster detection. This scan statistic, however, uses a circular window to define the potential cluster areas and thus has difficulty in correctly detecting actual noncircular clusters. A recent proposal by Duczmal and Assunção for detecting noncircular clusters is shown to detect a cluster of very irregular shape that is much larger than the true cluster in our experiences.

**Methods:**

We propose a flexibly shaped spatial scan statistic that can detect irregular shaped clusters within relatively small neighborhoods of each region. The performance of the proposed spatial scan statistic is compared to that of Kulldorff's circular spatial scan statistic with Monte Carlo simulation by considering several circular and noncircular hot-spot cluster models. For comparison, we also propose a new bivariate power distribution classified by the number of regions detected as the most likely cluster and the number of hot-spot regions included in the most likely cluster.

**Results:**

The circular spatial scan statistics shows a high level of accuracy in detecting circular clusters exactly. The proposed spatial scan statistic is shown to have good usual powers plus the ability to detect the noncircular hot-spot clusters more accurately than the circular one.

**Conclusion:**

The proposed spatial scan statistic is shown to work well for small to moderate cluster size, up to say 30. For larger cluster sizes, the method is not practically feasible and a more efficient algorithm is needed.

## Background

The question of whether disease cases are clustered in space has received considerable attention in the literature [1-4]. Although many statistical tests for disease clusters have been proposed, most tests suffer from multiple testing problems due to one or two unknown parameters that must be set prior to their applications. For example, Cuzick and Edwards's procedure [5] has an unknown number *k *of nearest-neighbours and Besag and Newell's method [6] has an unknown number of cases *k *for the size of the cluster. As far as we know, the spatial scan statistic proposed by Kulldorff [7,8] and Tango's maximized excess events test [9,10] are exceptions and take multiple testing into account in the sense that we have only to specify the maximum possible cluster size. Especially, Kulldorff's circular spatial scan statistic has been applied to a wide variety of epidemiological studies for cluster detection (for example, see [11-13]). In recent power comparisons of disease clustering tests, his scan statistic has been shown to be the most powerful for detecting localized clusters [14,15]. It should be noted, however, that the power estimates provided reflect the "power to reject the null hypothesis for whatever reason" and that the probability of both rejecting the null hypothesis and detecting the true cluster correctly is a different matter.

As the circular spatial scan statistic uses a "circular window" with variable size to define the potential cluster area, it is difficult to correctly detect noncircular clusters such as those along a river. Most geographical areas are noncircular. Furthermore, in our experience in applying SaTScan program [16] to various data, even if the null hypothesis is rejected, the circular spatial scan statistic tends to detect a larger cluster than the true cluster by absorbing surrounding regions where there is no elevated risk. It should be noted that although Kulldorff originally made no assumptions about the shape of the scanning window in his paper [8], a circular scanning window has been used in almost all purely spatial applications especially for the availability of software and computational speed.

Recently, Patil and Taillie [17] and Duczmal and Assunção [18] proposed non-circular spatial scan statistics based on the likelihood ratio test formulated in the same way as in the circular spatial scan statistic. To avoid undertaking computationally infeasible searches, they considered different approaches. Patil and Taillie [17] used the notion of "upper level set" to reduce the size of windows to be scanned and proposed "upper level set scan statistic". However, they do not discuss how to select the level *g *which defines the upper level set and do not provide any illustrations of their method nor any results of comparison with the circular scan statistic. Duczmal and Assunção [18], on the other hand, have applied a simulated annealing method in which they try to examine only the most promising windows using a graph-based algorithm to obtain the local maxima of a certain likelihood function over a subset of the collection of all the connected regions. Their method seems to be very complicated but they do not show any programmable procedure of their method. In our experience using their program (personal communication to Professor Duczmal via email) which is executable with the Borland C++ Builder 6, their scan statistic, in most cases, detected a cluster of peculiar shape that was much larger than the true cluster by absorbing not only surrounding regions with non-elevated risk but also faraway regions with non-elevated risk. An example of such properties of Duczmal and Assunção's procedure is shown later in comparison with the circular spatial scan statistic and the proposed flexible spatial scan statistic. That is why we did not include both the Patil and Taille method and Duczmal and Assunção's procedure in our simulation for comparison.

In this paper, we propose an alternative *flexibly shaped spatial scan statistic *('flexible spatial scan statistic' hereafter) in which the detected cluster is allowed to be flexible in shape while at the same time the cluster is confined within relatively small neighborhoods of each region. The performance of the flexible spatial scan statistic is compared with that of the circular spatial scan statistic using Monte Carlo simulation. In comparing performance we examined not only the usual power but also the newly introduced bivariate power distribution classified by the number of regions detected as the most likely cluster and the number of hot-spot regions included in the most likely cluster. The proposed flexible spatial scan statistic is illustrated with some simulated disease maps for the Tokyo Metropolitan area.

## Methods

Consider the situation where an entire study area is divided into *m *regions (for example, county, enumeration districts, etcetera). The number of cases in the region *i *is denoted by the random variable *N*_*i *_with observed value *n*_*i*_, *i *= 1, ..., *m*. Under the null hypothesis *H*_0 _of no clustering, the *N*_*i *_are independent Poisson variables such that

*H*_0 _: *E*(*N*_*i*_) = *ξ*_*i*_, *N*_*i *_~ Pois(*ξ*_*i*_), *i *= 1, ..., *m *    (1)

where Pois(*e*) denotes Poisson distribution with mean *e *and the *ξ*_*i *_are the null expected number of cases in the region *i*. To specify the geographical position of each region, we will use the coordinates of the administrative population centroid.

Under this situation, the circular spatial scan statistic imposes a circular window **Z **on each centroid. For any of those centroids, the radius of the circle varies from zero to a pre-set maximum distance *d *or a pre-set maximum number of regions *K *to be included in the cluster. If the window contains the centroid of a region, then that whole region is included in the window. In total, a very large number of different but overlapping circular windows are created, each with a different location and size, and each being a potential cluster. Let **Z**_*ik*_, *k *= 1,..., *K*, denote the window composed by the (*k *- 1)-nearest neighbours to region *i*. Then, all the windows to be scanned by the circular spatial scan statistic are included in the set

*Z*_1 _= {**Z**_*ik *_| 1 ≤ *i *≤ *m*, 1 ≤ *k *≤ *K*}     (2)

A flexible scan statistic we propose, on the other hand, imposes an *irregularly shaped *window **Z **on each region by connecting its adjacent regions. For any given region *i*, we create the set of irregularly shaped windows with *length k *consisting of *k *connected regions including *i *and let *k *moves from 1 to the pre-set maximum *K*. To avoid detecting a cluster of *unlikely peculiar shape*, the connected regions are restricted as the subsets of the set of regions *i *and (*K *- 1)-nearest neighbours to the region *i *where *K *is a pre-specified maximum length of cluster. In total, as in the circular spatial scan statistic, a very large number of different but overlapping arbitrarily shaped windows are created. Let **Z**_*ik*(*j*)_, *j *= 1,..., *j*_*ik *_denote the *j*-th window which is a set of *k *regions connected starting from the region *i*, where *j*_*ik *_is the number of *j *satisfying **Z**_*ik*(*j*) _⊆ **Z**_*ik *_for *k *= 1,..., *K*. Then, all the windows to be scanned are included in the set

*Z*_2 _= {**Z**_*ik*(*j*) _| 1 ≤ *i *≤ *m*, 1 ≤ *k *≤ *K*, 1 ≤ *j *≤ *j*_*ik*_}     (3)

In other words, for any given region *i*, the circular spatial scan statistic consider *K *concentric circles, whereas the flexible scan statistic consider *K *concentric circles plus all the sets of connected regions (including the single region *i*) whose centroids are located within the *K*-th largest concentric circle. So, the size of *Z*_2 _is far larger than that of *Z*_1 _which is at most *mK*. Details of the algorithm that we adopted to find all these arbitrarily shaped windows within a pre-specified maximum length *K *are given in the Appendix.

Under the alternative hypothesis, there is at least one window **Z **for which the underlying risk is higher inside the window when compared with outside. In other words, we are considering the following hypothesis:

*H*_0 _: *E*(*N*(**Z**)) = *ξ*(**Z**), for all **Z**, *H*_1 _: *E*(*N*(**Z**)) > *ξ*(**Z**), for some **Z **    (4)

where *N*() and *ξ*() denote the random number of cases and the null expected number of cases within the specified window, respectively. For each window, it is possible to compute the likelihood to observe the observed number of cases within and outside the window, respectively. Under the Poisson assumption, the test statistic, which was constructed with the likelihood ratio test [8], is given by



where **Z**^*c *^indicates all the regions outside the window **Z**, and *n*() denotes the observed number of cases within the specified window and *I*() is the indicator function. The window Z* that attains the maximum likelihood is defined as the *most likely cluster *(MLC). To find the distribution of the test statistic under the null hypothesis, Monte Carlo hypothesis testing [19] is required. In this paper, *p*-value of the test is based upon the null distribution of likelihood ratio test statistic with a large number (we used 999) of Monte Carlo replications of the data set generated under the null hypothesis. It should be noted that, in the same manner as the circular spatial scan statistic, the flexible spatial scan statistic is also able to locate secondary clusters that do not overlap the most likely cluster but are still statistically significant.

## Results

### Illustrations and powers

**Figure 1:**
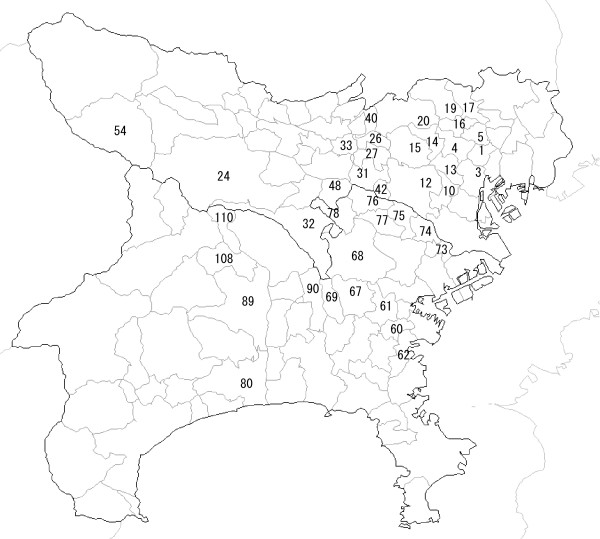
**An entire study population for simulation studies**. The 113 regions comprising wards, cities and villages in the area of Tokyo Metropolis and Kanagawa prefecture in Japan. The region number used in the text is shown. Especially, The region numbers of four hot-spot clusters **A-D **are **A **= {14, 15, 20}, **B **= {14, 15, 20, 26}, **C **= {14, 15, 26, 27}, and **D **= {73, 74, 75, 76, 78}, respectively.

In this section, we will compare the flexible spatial scan statistic with the circular spatial scan statistic. As an entire study population, we will use *m *= 113 regions comprising the wards, cities and villages in the area of Tokyo Metropolis and Kanagawa prefecture in Japan (Figure 1). The variability of regional populations for *m *= 113 regions is: 25 percentile = 56, 704, median = 142, 320 and 75 percentile = 200, 936.

#### Hot-spot clusters

We will consider the following four hot-spot clusters where the expected total number of cases  is set to be 200 under the null hypothesis.

1. Cluster **A **= {14, 15, 20}

2. Cluster **B **= {14, 15, 20, 26}

3. Cluster **C **= {14, 15, 26, 27}

4. Cluster **D **= {73, 74, 75, 76, 78}

where the region included in a hot-spot cluster is called a "hot-spot region" (hot-spot region numbers are shown in Figure 1). The relative risk within any cluster **R **is set to three, i.e.,

*H*_1 _: *N*(**R**) ~ Pois(*θξ*(**R**)), *θ *= 3.0     (6)

The cluster **A **is considered here as an example of a circular cluster that can be in the set of the circular windows and is expected to be identified by the circular spatial scan statistic more often than by the flexible spatial scan statistic. The other clusters are examples of noncircular clusters that are not in the set of the circular windows and thus cannot be identified correctly by the circular spatial scan statistics. For example, consider the region *i*_0 _= 15 as the starting region and the set of (*K *- 1)-nearest neighbours to the region 15, which is listed as follows in the ascending order of distance from the region 15:

15, 14, 20, 12, 4, 26, 13, 27, 16, 40, 19, 42, 10,...,

In this case, circular windows are {15}, {15, 14}, {15, 14, 20}, {15, 14, 20, 12}, ... When the starting region is 14 or 20, the corresponding set of (*K *- 1)-nearest neighbours is

14, 15, 20, 4, 16, 13, 19, 12, 5, 1, 17, 10, 26, 3, 27,...,

and

20, 14, 15, 19, 16, 4, 17, 26, 40, 13, 5, 12, 1, 27,...,

respectively. In both cases, cluster **B **and **C **are easily found to be not in the set of circular windows. The cluster **D **is considered as an example of a long and narrow cluster as is shown in Figure 1.

#### Illustrative example

**Figure 2:**
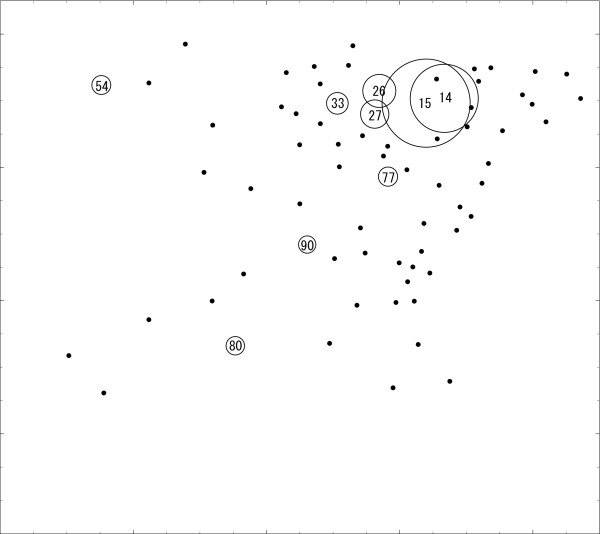
**A random sample from cluster model C**. Dots describe the centroids of regions with some cases. Circles are drawn only for the regions whose standardized risk ratios are statistically significantly larger than 1 at *α *= 0.05 and the region number is placed in stead of dot. The radius is set inversely proportional to the tail probability.

As an illustration, we will apply the circular spatial scan statistic, the flexible spatial scan statistic and Duczmal and Assunção's spatial scan statistic to the disease map shown in Figure 2 which is a random sample of *n *= 235 cases assuming the cluster model **C**. Circles are drawn only for the regions whose observed-expected ratio (standardized risk ratio) is statistically significantly larger than 1 at *α *= 0.05. The radius of the circles is set inversely proportional to the upper tail *p*-value. The number shown in Figure 2 indicates the region number. Figure 2 obviously suggests the clusters occurring in the area including regions {14, 15, 26, 27, 33}.

Before applying the three spatial scan statistics, we have to specify a common maximum length *K *for the most likely cluster. This makes comparisons to a certain extent fair. In this example, we chose two kinds of maximum length *K *= 15 and *K *= 20 since it is not unreasonable to assume that an actual cluster size will be less than one third or one fourth of the size of the whole study area.

**Figure 3:**
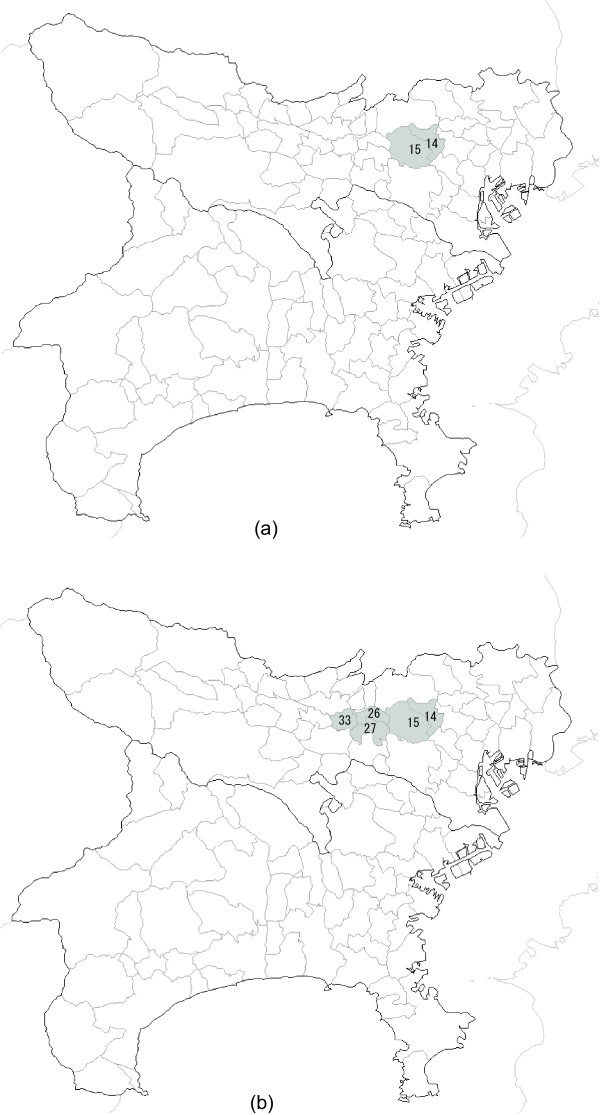
**The most likely cluster detected by the circular and the flexible spatial scan statistic**. (a) Detected by the circular spatial scan statistic for both *K *= 15 and *K *= 20 and (b) by the flexible spatial scan statistic for both *K *= 15 and *K *= 20, when applied to a random sample from the cluster model **C **= {14, 15, 26, 27}.

**Figure 4:**
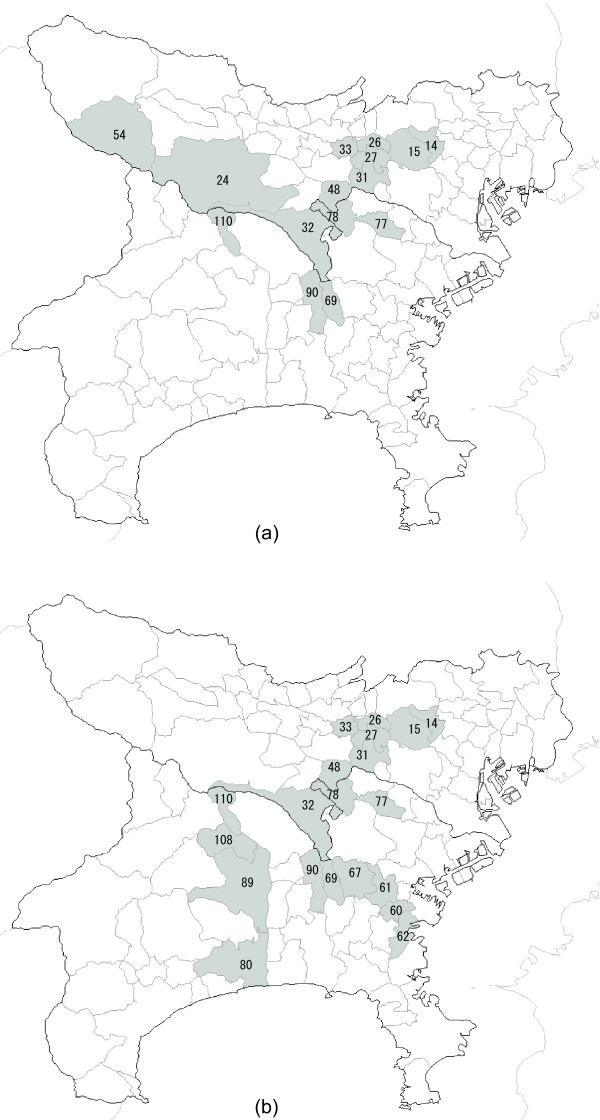
**The most likely cluster detected by the Duczmal and Assunção's scan statistic**. (a) Detected for *K *= 15 and (b) for *K *= 20, when applied to a random sample from the cluster model **C **= {14, 15, 26, 27}.

Irrespective of the value of *K*, the circular spatial scan statistic detected the regions {14, 15} as MLC with log likelihood ratio = 20.1, *p *= 1/(999 + 1) = 0.001 and the estimated relative risk is  = 3.47. This is shown in Figure 3(a). The flexible spatial scan statistic, regardless of the value *K*, detected the regions {14, 15, 26, 27, 33} as MLC with log likelihood ratio = 29.7, *p *= 0.001 and the estimated relative risk is  = 3.41. This is shown in Figure 3(b). Duczmal and Assunção's method, on the other hand, detected a cluster of peculiar shape that is much larger than the true cluster. In the case of *K *= 15, their scan statistic detected an area consisting of *K *= 15 connected regions {14, 15, 24, 26, 27, 31, 32, 33, 48, 54, 69, 77, 78, 90, 110 } as MLC with log likelihood ratio = 31.8, *p *= 0.001 and the estimated relative risk is  = 2.40. This is shown in Figure 4(a). Figure 4(b) shows the most likely cluster {14, 15, 26, 27, 31, 32, 33, 48, 60, 61, 62, 67, 69, 77, 78, 80, 89, 90, 108, 110 } detected by Duczmal and Assunção's scan statistic for *K *= 20 where the length of MLC is also the same as *K *= 20 and log likelihood ratio = 36.0, *p *= 0.001 and the estimated relative risk is  = 2.26. In the case of *K *= 15, the results of the three scan statistics are summarized in Table 1. Although the most likely cluster detected by Duczmal and Assunção's scan statistic has the largest log likelihood ratio among three scan statistics, it has detected MLC surprisingly larger than the true cluster.

**Table 1:** Regions detected as the most likely cluster by three procedures. Regions detected as the most likely cluster by the circular scan, the flexible scan and Duczmal and Assunção's scan, with the maximum length of cluster set to be *K *= 15 for the simulated random sample from the cluster model C where the hot spot cluster is assumed to be the set of connected four regions {14, 15, 26, 27} with the assumed relative risk *θ *= 3.0. For details, see text.

region no.	population	observed no. cases	expected no. cases	relative risk estimated (true)	Log likelihood ratio (LLR) and estimated relative risk for the most likely cluster		
					
					Circular	Flexible	Duczmal et al.
14	319,687	14	3.794	3.69 (3.0)	*	*	*
15	529,485	21	6.283	3.34 (3.0)	*	*	*
					LLR = 20.1		
					= 3.47		
26	139,077	6	1.650	3.64 (3.0)		*	*
27	165,564	6	1.964	3.05 (3.0)		*	*
33	105,899	4	1.257	3.18 (1.0)		*	*
						LLR = 29.7	
						= 3.41	
24	466,347	8	5.534	1.44 (1.0)			*
31	197,677	3	2.346	1.27 (1.0)			*
32	349,050	5	4.142	1.20 (1.0)			*
48	58,635	1	0.696	1.43 (1.0)			*
54	3,808	1	0.045	22.12(1.0)			*
69	119,575	3	1.419	2.11 (1.0)			*
77	177,742	5	2.109	2.37 (1.0)			*
78	125,127	2	1.485	1.34 (1.0)			*
90	194,866	5	2.312	2.16 (1.0)			*
110	21,535	1	0.256	3.91 (1.0)			*
							LLR = 31.8
							= 2.41

Using a PC(Windows XP, CPU pentium 4, 3.2 GHz), the execution time of the flexible spatial scan statistic in this example is 14 seconds for *K *= 15 and 379 seconds for *K *= 20 which is certainly greater than that for the circular spatial scan statistic (less than 1 second for both *K *= 15 and *K *= 20).

#### Power comparison

In the power comparison, we chose *K *= 15. To compare the power of the flexible spatial scan statistic with that of the circular spatial scan statistic based upon Monte Carlo simulation, we will introduce a new bivariate power distribution *P*(*l*, s) classified by the *length l *of the significant MLC and the number *s *of hot-spot regions included in the most likely cluster:



where *l *≥ 1 and *s *≥ 0. Based on *P*(*l*, *s*), we examined the following powers,

1. the usual power, i.e., *P*(*+,+*) = ∑_*l*≥1 _∑_*s*≥0 _*P*(*l*, *s*),

2. the joint power *P*(*l*, *s*), especially *P*(s*, s*) where s* is the length of the hot-spot cluster assumed in the simulation.

3. the marginal power distribution of *s*(≥ 0), *P*(+, *s*) = ∑_*l*≥1 _*P*(*l*, *s*) and its conditional power *P*(+, *s*)/*P*(+,+),

4. the marginal power distribution of *l*(≥ 1), *P*(*l*, +) = ∑_*s*≥0 _*P*(*l*, *s*).

The powers are calculated for tests of nominal *α *levels of 0.05 and for the expected total number of cases 200 under the null hypothesis, which are based on Monte Carlo simulation using Poisson random numbers. For each simulation, 1,000 trials were carried out. The resultant power distribution *P*(*l*, *s*) × 1000 is shown in Tables 2, 3, 4, 5 for each of the four cluster models, respectively, in the form of cross table classified by *l *(*"length" *in tables) and *s *(*"include" *in tables).

**Table 2:** Comparison of the circular and the flexible spatial scan statistic for the cluster model A. Comparison of bivariate power distribution *P*(*l*, *s*) × 1000 between the circular spatial scan statistic and the flexible spatial scan statistic for the hot-spot cluster **A **= {14, 15, 20}. Nominal *α*-level is set as 0.05 and 1000 trials are carried out. For more details, see text.

Flexible (*K *= 15)						Circular (*K *= 15)					
	
Length *l*	Include *s * hot-spot regions				Total	Length *l*	Include *s * hot-spot regions				Total
					
	0	1	2	3			0	1	2	3	
	
1	0	0			0	1	0	0			0
2	0	0	0		0	2	1	0	0		1
3	0	0	0	142	142	3	0	0	0	738	738
4	0	0	0	116	116	4	0	0	0	134	134
5	0	0	0	137	137	5	0	0	0	39	39
6	0	0	0	149	149	6	0	0	0	12	12
7	0	0	0	165	165	7	0	0	0	9	9
8	0	0	0	131	131	8	0	0	0	1	1
9	0	0	0	84	84	9	0	0	2	3	5
10	0	0	0	27	27	10	0	0	0	2	2
11	0	0	0	11	11	11	0	0	0	4	4
12	0	0	0	2	2	12	0	0	0	12	12
13	0	0	0	0	0	13	0	0	0	14	14
14	0	0	0	0	0	14	0	0	0	3	3
15	0	0	0	0	0	15	0	0	0	6	6
	
Total	0	0	0	964	964	Total	1	0	2	977	980
	usual power = 0.964						usual power = 0.980				

**Table 3:** Comparison of the circular and the flexible spatial scan statistic for the cluster model B. Comparison of bivariate power distribution *P*(*l*, *s*) × 1000 between the circular spatial scan statistic and the flexible spatial scan statistic for the hot-spot cluster **B **= {14, 15, 20, 26}. Nominal *α*-level is set as 0.05 and 1000 trials are carried out. For more details, see text.

Flexible (*K *= 15)							Circular (*K *= 15)						
	
Length *l*	Include *s * hot-spot regions					Total	Length *l*	Include *s * hot-spot regions					Total
					
	0	1	2	3	4			0	1	2	3	4	
	
1	0	0				0	1	0	0				0
2	0	0	0			0	2	0	0	0			0
3	0	0	0	0		0	3	0	0	0	523		523
4	0	0	0	0	127	127	4	0	0	0	65	0	65
5	1	0	0	0	157	158	5	0	0	0	23	0	23
6	0	0	0	0	205	205	6	0	0	0	7	66	73
7	0	0	0	2	198	200	7	0	0	0	0	15	15
8	0	0	0	1	151	152	8	0	0	0	0	32	32
9	0	0	0	5	85	90	9	0	0	0	1	15	16
10	0	0	0	1	24	25	10	0	0	0	0	7	7
11	0	0	0	0	17	17	11	0	0	0	2	3	5
12	0	0	0	0	5	5	12	0	0	0	2	63	65
13	0	0	0	0	0	0	13	0	0	0	0	96	96
14	0	0	0	0	0	0	14	0	0	0	0	30	30
15	0	0	0	0	0	0	15	0	0	0	0	22	22
	
Total	1	0	0	9	969	979	Total	0	0	0	623	349	972
	usual power = 0.979							usual power = 0.972					

**Table 4:** Comparison of the circular and the flexible spatial scan statistic for the cluster model C. Comparison of bivariate power distribution *P*(*l*, *s*) × 1000 between the circular spatial scan statistic and the flexible spatial scan statistic for the hot-spot cluster **C **= {14, 15, 26, 27}. Nominal *α*-level is set as 0.05 and 1000 trials are carried out. For more details, see text.

Flexible (*K *= 15)							Circular (*K *= 15)						
	
Length *l*	Include *s * hot-spot regions					Total	Length *l*	Include *s * hot-spot regions					Total
					
	0	1	2	3	4			0	1	2	3	4	
	
1	0	0				0	1	1	0				1
2	0	0	0			0	2	0	0	351			351
3	0	0	0	0		0	3	2	0	4	0		6
4	0	0	0	0	138	138	4	0	0	3	0	0	3
5	0	0	0	3	147	150	5	2	0	2	0	0	4
6	1	0	0	2	200	203	6	1	0	0	0	0	1
7	0	1	0	4	147	152	7	0	0	0	81	0	81
8	0	0	2	9	107	118	8	0	0	10	18	38	66
9	0	0	0	10	71	81	9	0	0	2	0	26	28
10	1	0	2	5	28	36	10	0	0	0	29	3	32
11	0	0	0	0	10	10	11	0	0	1	13	1	15
12	0	0	0	0	2	2	12	0	0	2	4	60	66
13	0	0	0	0	0	0	13	0	0	0	5	62	67
14	0	0	0	0	0	0	14	0	0	0	10	27	37
15	0	0	0	0	0	0	15	0	0	0	6	37	43
	
Total	2	1	4	33	850	890	Total	6	0	375	166	254	801
	usual power = 0.890							usual power = 0.801					

**Table 5:** Comparison of the circular and the flexible spatial scan statistic for the cluster model D. Comparison of bivariate power distribution *P*(*l*, *s*) × 1000 between the circular spatial scan statistic and the flexible spatial scan statistic for the hot-spot cluster **D **= {73, 74, 75, 76, 78}. Nominal *α*-level is set as 0.05 and 1000 trials are carried out. For more details, see text.

Flexible (*K *= 15)								Circular (*K *= 15)							
	
Length *l*	Include *s * hot-spot regions						Total	Length *l*	Include *s * hot-spot regions						Total
					
	0	1	2	3	4	5			0	1	2	3	4	5	
	
1	0	0					0	1	6	0					6
2	1	0	0				1	2	3	5	0				8
3	0	0	0	0			0	3	0	0	0	14			14
4	1	0	0	1	0		2	4	1	0	4	5	0		10
5	0	1	0	3	1	242	247	5	0	0	2	1	0	0	3
6	1	0	0	1	2	162	166	6	1	0	0	1	363	0	365
7	2	3	0	5	5	93	108	7	0	0	1	0	56	0	57
8	1	2	1	6	7	53	70	8	0	0	2	2	28	0	32
9	0	2	0	1	5	38	46	9	0	0	2	2	10	0	14
10	0	2	0	1	1	18	22	10	1	0	0	3	3	0	7
11	0	0	0	2	2	5	9	11	0	0	0	0	3	11	14
12	0	0	1	0	0	1	2	12	0	0	0	2	3	8	13
13	0	0	0	0	0	0	0	13	0	0	0	1	1	16	18
14	0	0	0	0	0	0	0	14	0	0	1	0	0	5	6
15	0	0	0	0	0	0	0	15	0	1	0	0	1	7	9
	
Total	6	10	2	20	23	612	673	Total	12	6	12	31	468	47	576
	usual power = 0.673								usual power = 0.576						

##### 1) Usual power

Both tests have the same size 0.043 (distribution of length of significant MLC is omitted) and are shown to have high powers for the hot-spot clusters considered here. The flexible spatial scan statistic generally has higher power except for the model **A **(circular cluster) where, however, the difference is small.

##### 2) Joint powers at (s*, s*) and at its neighbours

Table 2 shows the good characteristics of the circular spatial scan statistic. Namely, the circle-based scan statistic could detect circular hot-spot cluster **A **with length s* = 3 considerably more accurately with power 738/1000 compared to 142/1000 of the flexible spatial scan statistic. Tables 3, 4, 5, on the other hand, show that the power of the circular spatial scan statistic in detecting exactly noncircular hot-spot clusters is 0/1000 due to the circular window. However, the circular spatial scan statistic is seen to be able to include some of the hot-spot regions into MLC reasonably well. For example, when applied to the noncircular cluster **B **with length s* = 4, three or four regions including three hot-spot regions can be detected as the most likely cluster with relatively high power (523 + 65)/1000 = 0.588 (Table 3). When applied to the model **D **with length s* = 5, the similar high power 363/1000 can be observed at (*l*, *s*) = (6, 4) (Table 5). The flexible spatial scan statistic, on the other hand, has no such high power at a single point (*l*, *s*) near (s*, s*). However, the characteristic of the flexible spatial scan statistic is that the support of the power distribution is distributed in a relatively narrow range of / on the line *s *= s*,i.e, we have s* ≤ *l *≤ 12 in the four cluster models considered here.

##### 3) Marginal power *P*(+, *s*) and its conditional marginal power *P*(+, *s*)/*P*(+, +)

Regarding the marginal power *P*(+, s*) at *s *= s*, the flexible spatial scan statistic is shown to have much higher power than the circular spatial scan statistic for the case of noncircular clusters (Tables 3, 4, 5). Furthermore, the conditional marginal power *P*(+, *s*)/*P*(+, +) of the flexible spatial scan statistic is 964/964 = 1.000, 969/979 = 0.990, 850/890 = 0.955 and 612/673 = 0.909 for the cluster **A-D**, respectively. These results indicate that the identified MLC by the flexible spatial scan statistic includes the hot-spot cluster with quite high probability. For the noncircular clusters, the mode of *P*(+, *s*) of the circular spatial scan statistic is around *s *= s* - 1 or *s *= s* - 2.

##### 4) Marginal power distribution *P*(*l*, +)

For the flexible spatial scan statistic, the probability that the length of significant MLC is less than *s *= s* is shown to be zero or quite small and the maximum length is around 10 to 12. the circular spatial scan statistic, on the other hand, tends to detect a much longer cluster than expected from the hot-spot cluster assumed in the simulation. For example, the probability that the length of MLC for the cluster B with length s* = 4 is greater than or equal to 12 is 213/1000 = 0.213 compared with 5/1000 = 0.005 for the flexible spatial scan statistic. The probability that the length of MLC for the cluster **C **with length s* = 4 is greater than or equal to 12 is 213/1000 = 0.213 compared with 2/1000 = 0.002 for the flexible spatial scan statistic. This tendency is shown even in the circular cluster **A **where the same probabilities are 0.035 vs. 0.002.

#### Cost comparison

Based upon the bivariate power function *P*(*l*, *s*), we can compute the following expected total cost incurred by incomplete identification of the true cluster:

*C *= *C*_2_{*rE*(s* - *S*) + *E*(*L *- *S*)}, *r *= *C*_1_/*C*_2 _    (8)

where *C*_1 _and *C*_2 _denote the average cost of missing one region in the true cluster and that of incorrectly detecting one region not in the true cluster, respectively. *L *and *S *denote the random variable of *l *and *s*, respectively. Two expected numbers *E*(s* - *S*) and *E*(*L *- *S*) for four kinds of clusters **A-D **are shown in Table 6. In general, we can assume *r *> 1. For example, the ratio *C*/*C*_2 _is shown for the case of *r *= 1 and *r *= 2, respectively, in Table 6. However, in this example, irrespective of the value of *r*(> 1), the circular spatial scan statistic is shown to have lower cost for detecting circular cluster **A **but to have higher cost for detecting non-circular clusters **B-D**.

**Table 6:** Cost comparison Expected number of undetected regions included in the true cluster *E*(s* - *S*), expected number of detected regions not in the true cluster *E*(*L *- *S*) and the ratio of costs *C*/*C*_2 _(*r *= 1, 2) incurred by incomplete identification of the true cluster. The spatial scan statistic with low values is better.

Hot-spot Cluster	Scan statistic	*E*(s* - *S*)	*E*(*L *- *S*)	the ratio *C*/*C*_2_	
				
				*r *= 1	*r *= 2
**A **= {14, 15, 20}	Flexible (K = 15)	0.108	2.951	3.059	3.167
	Circular (K = 15)	0.065	0.722	0.787	0.852
					
**B **= {14, 15, 20, 26}	Flexible (K = 15)	0.097	2.548	2.645	2.742
	Circular (K = 15)	0.735	2.525	3.260	3.995
					
**C **= {14, 15, 26, 27}	Flexible (K = 15)	0.492	2.243	2.735	3.227
	Circular (K = 15)	1.736	3.153	4.889	6.625
					
**D **= {73, 74, 75, 76, 78}	Flexible (K = 15)	1.774	1.088	2.862	4.636
	Circular (K = 15)	2.770	1.709	4.479	7.249

### Limitations of current work

Needless to say, the results derived here are based upon a small Monte Carlo simulations study and thus the characteristic observed in the current work could change a little bit depending on the cluster model adopted. We assumed here only one hot spot cluster and did not consider the case of several hot spot clusters. Therefore, we need a further simulation study to compare the performance of the two spatial scan statistics under several different clusters.

Regarding the algorithm adopted for the flexible spatial scan statistic, we set the restriction that irregularly shaped windows **Z **with length *k*(≤ *K*) are constructed from members of the (*K *- 1)-nearest neighbours to the starting region. It seems that this restriction plays an important role in preventing the flexible spatial scan statistic from reaching out for and absorbing faraway regions with non-elevated risk. However, to avoid undertaking computationally infeasible searches, the flexible spatial scan statistic has to be set with an upperbound for *K*. This depends on the disease map under study and the capability of the computer. The current practical upperbound is around *K *= 30 for the reason that the execution time of our current algorithm will take more than a week if *K > *30 for the number of regions *m *= 200 ~ 300. However, it seems to be unlikely that the length of the true cluster would be larger than 10 ~ 15 percent of the total number of regions. So, we think that our current algorithm can be applied to many epidemiological studies with small to moderate cluster sizes. However, for larger cluster sizes, a more sophisticated algorithm to increase the upperbound for *K *is needed.

Regarding data type, the proposed spatial scan statistic can only be applied to regional count data whereas the circular spatial scan statistic can be applied to not only count data but also individual point data. However, at least in disease surveillance, most of the data that people analyze is aggregated, so the method covers most real-world situations.

Finally, one of the reviewers commented that using small areas as basis for clustering without any attempt to incorporate heterogeneity in background rates is a fundamental flaw of all existing scanning methods. In general, we know that disease risks over study regions are heterogeneous to a certain extent and the null hypothesis of complete spatial randomness is not true. However, statistical hypothesis testing is based upon the null hypothesis which is not true. Likewise, we will use complete spatial randomness as the null hypothesis as indicated in equation (1) since we are interested in rejecting the null hypothesis and detecting the local clusters with excess risk. If we are interested in estimating a clustering mechanism, we should use some modeling approach rather than spatial scan statistics.

## Discussion

In this paper, we proposed a flexibly shaped spatial scan statistic to detect arbitrarily shaped clusters by amalgamating administrative units. The flexible spatial scan statistic is, via Monte Carlo simulation, shown to have reasonably high powers compared with the circular spatial scan statistic when examined by a newly introduced bivariate power distribution *P*(*l*, *s*). The simulation reveals that the circular spatial scan statistics shows a high level of accuracy in detecting circular clusters exactly and reasonably good power for including some hot-spot regions into the most likely cluster. The flexible spatial scan statistic exhibits no such high power regarding exact identification of clusters but the support of the power distribution is shown to be concentrated in a relatively narrow range of length *l *on the line *s *= s*, indicating that an observed significant most likely cluster contains the true cluster with quite high probability. The circular spatial scan statistic, on the other hand, is shown to have zero powers for detecting exactly noncircular clusters that cannot be captured by any circular window. The circular spatial scan statistic is also shown to have a tendency to detect a larger cluster than the true cluster assumed in the simulation even for the case when the true cluster is circular. Furthermore, by introducing the two kinds of cost due to incomplete detection of the true cluster, we could summarize these characteristics in terms of minimizing expected total cost. One of the reviewers suggested a similar cost comparison using the number of people that are incorrectly classified rather than the number of regions since the cost of misclassifying a large region is at least for disease surveillance purposes higher than that of misclassifying a region with smaller population. We think that would be an interesting additional simulation study worth conducting. However, since it can be expected that the result of such a cost comparison strongly depends on the spatial configuration of regions with different population size in the neighborhood of and within the true cluster and thus it requires careful design for creating suitable cluster models from which we can intuitively infer the result to a certain extent, we would like to leave such a simulation study in our future work.

The surprising result that Duczmal and Assunção's scan statistic detected quite large and unlikely peculiar shaped clusters that had the largest likelihood ratio among the three scan statistics might cast a doubt on the validity of the model selection based upon maximizing the likelihood ratio (5). Such a doubt can also be seen in some simulation results of the circular spatial scan statistic that had non-negligible probabilities of detecting much longer clusters than the true cluster. The flexible spatial scan statistic, on the other hand, is shown not to detect such an unexpected long cluster probably because it has the restriction that our windows are constructed only from members of the (*K *- 1)-nearest neighbours to the starting region. Nevertheless, these undesirable properties produced by maximum likelihood ratio might suggest the use of a different criterion for model selection. For example, we might consider a penalized likelihood where we consider a penalty for the *complexity of the cluster shape*, which is also worth future research.

All the computations and simulations have been conducted on a PC with Windows XP. For users who are interested in applying the flexible spatial scan statistic, we can provide the software FleXScan [20].

## Conclusion

The circular spatial scan statistics shows a high level of accuracy in detecting circular clusters exactly and reasonably good power for including some hot-spot regions into the most likely cluster. The proposed flexible spatial scan statistic is shown to have good usual powers plus the ability to detect the noncircular hot-spot clusters more accurately than the circular spatial scan statistic. However, the proposed spatial scan statistic work well for small to moderate cluster size, say up to 30. For larger cluster sizes, the method is not practically feasible and a more efficient algorithm is needed.

## Appendix: algorithm to find the set *Z*_2 _defined in equation (3)

There are probably several procedures to find the set *Z*_2 _that is defined as the set of arbitrarily shaped windows **Z **within a pre-specified maximum length *K*. The algorithm that we used is described as follows:

Step 1. First we set an *m *× *m *matrix *A *= (*a*_*ij*_) such as



and set *Z*_2 _= *φ *and *i*_0 _= 0

Step 2. Let *i*_0 _← *i*_0 _+ 1 and *i*_0_(= 1, 2,..., *m*) be the starting region. Then we create the set  consisting of (*K *- 1)-nearest neighbours to the starting region *i*_0 _and *i*_0 _itself, i.e.,

 = {*i*_0_, *i*_1_, *i*_2_,..., *i*_*K *- 1_},

where *i*_*k *_is the *k*-th nearest to *i*_0_.

Step 3. We consider all the set **Z **⊂ , which includes the starting region *i*_0_. For any given such set **Z**, repeat the following steps 4–7.

Step 4. We divide the set **Z **into two disjoint sets: **Z**_0 _= {*i*_0_} and **Z**_1 _which contains the other regions of **Z**.

Step 5. We make two new sets  and .  consists of the regions of **Z**_1 _that are connected to some regions of **Z**_0_. On the other hand,  consists of the regions of **Z**_1 _that are not connected to any regions of **Z**_0_. Then we replace **Z**_0 _and **Z**_1 _by  and , respectively.

Step 6. We repeat the step 5 recursively until either **Z**_0 _or **Z**_1 _becomes null first.

Step 7. We make a decision as follows. **Z **is said to be "connected" when **Z**_1 _becomes null first and "disconnected" when **Z**_0 _becomes null first. If we can find **Z **"connected", **Z **is added to the set *Z*_2_. If we find **Z **"disconnected", **Z **is discarded.

Step 8. Repeat the steps 2–7 until we finally get the set *Z*_2 _consisting of arbitrarily shaped windows **Z **whose maximum length is *K*.

Now we shall give an example using regions in the Tokyo Metropolitan area shown in Figure 1. Let the starting region *i*_0 _= 14. Then, the regions in the set of (*K *- 1)-nearest neighbours to the region 14 are listed as follows in the ascending order of distance to the region 14, i.e.,

**W**_14 _= {14, 15, 20, 4, 16, 13, 19, 12, 5,...}.

Suppose that we take a subset **Z **= {14, 15, 20, 26}. In the first step, we have

**Z**_0 _= {14}, **Z**_1 _= {15, 20, 26}.

Since *a*_14,15 _= *a*_14,20 _= 1 and *a*_14,26 _= 0, we then have

**Z**_0 _= {15, 20}, **Z**_1 _= {26}.

Further, because *a*_15,26 _= *a*_20,26 _= 1, these sets are replaced by

**Z**_0 _= {26}, **Z**_1 _= *φ*.

So, we can find that the set **Z **= {14, 15, 20, 26} is "connected" and can be a member of *Z*_2_.

If we take a subset **Z **= {14, 15, 20, 5}, we can find **Z **is "disconnected" because *a*_14,5 _= *a*_15,5 _= *a*_20,5 _= 0, **Z**_0 _= *φ *and **Z**_1 _= {5} at the final stage.

## Authors' contributions

TT proposed the flexibly shaped spatial scan statistic and the bivariate power distribution. KT considered the algorithm given in the appendix, programmed the C++ code and carried out the power simulations. TT wrote the first draft of the manuscript. Both authors interpreted the results and wrote the final version of the paper.
